# Autologous Serum Skin Test versus Autologous Plasma Skin Test in Patients with Chronic Spontaneous Urticaria

**DOI:** 10.1155/2013/267278

**Published:** 2013-07-09

**Authors:** Aysegul Alpay, Nilgün Solak Tekin, Ishak Özel Tekin, H. Cevdet Altinyazar, Rafet Koca, Saniye Çınar

**Affiliations:** ^1^Bulent Ecevit University, School of Medicine, Department of Dermatology, Kozlu, 67600 Zonguldak, Turkey; ^2^Bulent Ecevit University, School of Medicine, Department of Immunology, Turkey; ^3^Selcuk University, Selcuklu Medical Faculty, Department of Dermatology, Turkey

## Abstract

Previous studies indicate that 25–45% of chronic urticaria patients have an autoimmune etiology. Autologous serum skin test (ASST) and autologous plasma skin test (APST) are simple tests for diagnosing chronic autoimmune urticaria (CAU). However, there are still some questions about the specificity of these tests. This study consisted of 50 patients with chronic spontaneous urticaria (CSU) and 50 sex- and age-matched healthy individuals aged 18 years, and older. A total of 31 (62%) patients and 5 (10%) control patients had positive ASST; 21 (42%) patients and 3 (6%) control patients had positive APST. Statistically significant differences were noted in ASST and APST positivity between the patient and control groups (ASST *P* < 0.001; APST *P* < 0.001). Thirteen (26%) patients and 5 (10%) control patients had antithyroglobulin antibodies or antithyroid peroxidase antibody positivity. No statistically significant differences were noted in thyroid autoantibodies between the patient and control groups (anti-TG *P* = 0.317; anti-TPO *P* = 0.269). We consider that the ASST and APST can both be used as *in vivo* tests for the assessment of autoimmunity in the etiology of CSU and that thyroid autoantibodies should be checked even when thyroid function tests reveal normal results in patients with CSU.

## 1. Introduction

Chronic spontaneous urticaria is a skin disorder which follows a course with erythematous, edematous, and itchy plaques, almost daily, for over 6 weeks and which is associated with lower quality of life affecting work and daily activities of patients [1, 2]. The most important causes of chronic spontaneous urticaria include chronic infections, food intolerance, chronic noninfectious inflammatory diseases (gastritis, esophagitis, etc.), and atopy [3]. No apparent cause is identifiable in approximately 70% of patients [4–7]. This patient group, termed chronic idiopathic urticaria, is subdivided into “chronic autoimmune urticaria (CAU)” and “chronic spontaneous urticaria (CSU)”, and CAU accounts for 25% of patients with chronic urticaria [6, 8]. There is sound evidence indicating the presence of functional histamine releasing autoantibodies or nonantibody factors (serum factor) against the *α* subunit (Fc*ε*RI*α*) of IgE or the high affinity IgE receptors from basophil and mast cells in patients with CAU [5, 6, 9–11]. The presence of these autoantibodies is demonstrated by papular and erythematous reaction by intradermal injection of autologous serum or autologous plasma (autologous serum skin test (ASST); autologous plasma skin test (APST)) [12–14]. ASST is an *in vivo* test used for the diagnosis of autoimmune urticaria since 1940, and a positive reaction is detected during the active phases of the disease [15]. Recently, Asero et al. have reported that the APST is more sensitive than the ASST but cannot be considered as a screening test for histamine-releasing autoantibodies [16]. However, there are few studies on this issue in the literature.

There are a number of studies supporting the relationship between thyroid autoimmunity and urticaria [17, 18]. The presence and severity of chronic urticaria in patients with autoimmune thyroiditis have been shown to be significantly higher than that in patients with negative thyroid autoantibodies and healthy controls [2, 19, 20]. The objective of this study was to compare the ASST and APST used for the assessment of autoimmunity in patients with CSU in terms of efficacy and feasibility and to evaluate their relationship with thyroid autoantibodies, thus enabling selection of the most appropriate treatment protocol for each patient.

## 2. Materials and Methods

### 2.1. Patients

Fifty patients with clinically definite chronic spontaneous urticaria, and 50 sex- and age-matched healthy controls were included in this prospective study. The study was approved by the Local Ethics Committee, and informed written consent was obtained from all patients and volunteers before study entry. A detailed medical history was obtained from each patient and routine laboratory tests (hemogram, total IgE, etc.), and physical examinations were performed for all patients. In all cases, known causes of urticaria, such as food allergy, additive in tolerance, chronical infections such as dental infections or *Helicobacter pylori* infections, and parasitoses or systemic diseases, had been excluded by appropriate investigations. Patients with physical urticaria were not included in the study. A majority of the patients enrolled in this study had received at least one antihistamine and/or mast cell stabilizer previously and received no benefit from the treatment for a long time. None of the patients had received immunosuppressive therapy previously, except for a short-term systemic corticosteroid therapy given during acute attacks.

### 2.2. Skin Tests

All patients in the patient and control groups underwent ASST and APST as well as testing for antithyroglobulin antibodies (anti-TG) and antithyroid peroxidase antibodies (anti-TPO). All patients were required to withold antihistamines for at least 72 hours and mast cell stabilizers for at least 1 week before the ASST and APST. Prior to the test, adrenaline, injectable methylprednisolone and antihistamines were kept ready for the possibility of an anaphylactic reaction. For the ASST, 5 cc samples of venous blood were obtained from all patients and healthy controls into sterile glass tubes. For the APST, 5 cc samples of venous blood were taken into sterile glass tubes containing 0.125 mol/L sodium citrate, and the blood samples were kept at room temperature for 30 minutes. Then, the blood samples were separated into serum and plasma by centrifugation at 2500 rpm (Nuve NF 1200). In the active phase of the disease, 0.1 mL of undiluted autologous serum, 0.1 mL of autologous plasma, and 0.1 mL of saline solution were injected intradermally using a 30-gauge needle, keeping a distance of 5 cm between the injection sites, over the volar aspect of the right forearm, avoiding the areas of urticarial papules within the past 24 hours. Histamine phosphate, used as a positive control, was injected on the volar aspect of the left forearm as a prick test. Test results were measured at 30 min. The ASST and APST were considered positive when an erythematous papule induced by autologous serum and autologous plasma with a mean diameter more than 1.5 mm or more than that of the saline induced response was present. 

### 2.3. Statistics

The measurement of thyroid autoantibodies was performed using the Immulite 2000 system. Statistical analysis was performed using SPSS version 13.0. The Kolmogorov-Smirnov test was used to evaluate the normal distribution of numerical variables. Descriptive statistics for numerical variables are presented as mean ± standard deviation, whereas categorical data are presented as number and percentage. The Chi-square and Fisher-exact chi squared tests were used to analyze the differences between the groups in terms of categorical variables and the relationships between the variables. Comparison of means between two groups was performed using significance test of the difference between two means when the assumptions of parametric test were met and using the Mann-Whitney *U* test when the assumptions of parametric test were not met. A *P* value of <0.05 was considered statistically significant with a confidence interval of 95%.

## 3. Results

A total of 11 (22%) male patients and 39 (78%) female patients with a mean age of 46.1 ± 13.5 and 9 (18%) male patients and 41 (82%) female healthy individuals with a mean age of 45.1 ± 9.5 were enrolled in this study. The patient and control groups were statistically similar in age and sex distributions (mean age *P* = 0.671; sex *P* = 0.803). There were no statistically significant differences in thyroid autoimmunity and thyroid function between the patient and control groups (Table 1).

A total of 31 (62%) patients had positive ASST and 19 (38%) patients had negative ASST whereas 21 (42%) patients had positive APST and 29 (58%) patients had negative APST. Fourteen patients had positive ASST and APST and 12 patients had negative ASST and APST. Seventeen patients with positive ASST had negative APST whereas 7 patients with positive APST had negative ASST.

In the control group, 5 (10%) patients had positive ASST and 45 (90%) patients had negative ASST. The APST was positive in 3 (6%) of the healthy individuals and negative in 47 (94%). ASST and APST positivity were statistically significant between the patient and control groups. ASST and APST positivity were significantly higher in the patient group compared to the control group (ASST *P* < 0.001; APST *P* < 0.001). No statistically significant differences were noted between the results of ASST and APST in the patient and control groups (Tables 2-3). The compatibility of these two *in vivo* tests, which are used in the diagnosis of chronic autoimmune urticaria, was evaluated with Kappa test. And the relation between the two was found significant. In our paper, the tests were found to be %30 percent compatible with each other. 

No statistically significant differences were noted in the duration of disease, duration of papules, interval between attacks, and the association of angioedema between the patients (Table 4).

The size of erythematous papule was measured as between 6–20 mm (median: 11) diameter for ASST and 5–13 mm (median: 8) for APST in patient group. The diameter of erythematous papule was measured as between 5–11 mm (median: 6) for ASST and 5–10 mm (median: 6) for APST in control group.

Three patients with positive ASST were positive for anti-TG antibodies, and anti-TPO antibodies and these patients were functionally euthyroid. No statistically significant differences were observed in thyroid autoimmunity between the patients with positive ASST and those with negative ASST (*P* = 0.695 for anti-TG; *P* = 1.000 for anti-TPO). There were statistically significant differences in the relationship between thyroid function (TSH) and ASST positivity and negativity in patients with chronic urticaria (*P* = 0.049). Euthyroid patients with chronic urticaria had a higher rate of ASST positivity. Three patients with positive APST were positive for anti-TG and anti-TPO antibodies, and these patients were functionally euthyroid. No statistically significant differences were observed in thyroid autoimmunity and TSH between patients with positive or negative APST (*P* = 0.434 for anti-TG; *P* = 0.686 for anti-TPO; *P* = 0.254 for thyroid function).  Table 5 presents the relationship between thyroid autoantibodies and ASST and APST responses in patients with chronic urticaria.

There were no statistically significant differences in thyroid autoimmunity and TSH between healthy individuals with positive or negative ASST and APST (Table 6).

## 4. Discussion

The “*in vitro* basophil histamine release test” remains the gold standard for the detection of functional autoantibodies in patients with chronic autoimmune urticaria. In this test, human basophils from two healthy donors are stimulated with patient serum, and the subsequent histamine release is measured, and the presence of circulating functional autoantibodies is detected [21]. However, this test is not widely used because it requires fresh basophils from healthy donors, is time consuming, and may miss nonfunctional autoantibodies [5]. The measurement of the expression of CD63 and CD203c by donor basophils after incubation with patient serum has been described as a reliable novel method [22]. The modified CD63 expression assay was found to be a reliable method for the diagnosis of CAU [23]. The ELISA and Western blot analysis can also be used to detect the presence of autoantibodies; however, they cannot differentiate between functional and nonfunctional autoantibodies and take a long time to be completed [21].

The development of erythema and swelling following intradermal injection of autologous serum in patients with CIU is the first indicator of the presence of circulating autoantibodies in some patients. This observation provides the basis of ASST used for the diagnosis of CAU [21]. The demonstration of autoantibodies against *in vitro* IgE and Fc*ε*RI by immunological methods in only some patients with positive ASST and APST (25–50%) confirms that factors other than autoantibodies play a role in the etiology [24]. For this reason, a positive ASST is suggestive of autoimmunity; however, the presence of antibodies should be confirmed by *in vitro* tests [25]. The prevalence of a positive ASST result ranges from 50% to 60% in patients with CSU [15]. Thus, the ASST is regarded as a simple and cost effective *in vivo* test that contributes considerably to elucidation of the pathogenesis of chronic urticaria and can demonstrate the presence of functional autoantibodies in parallel to *in vitro* basophil histamine release [21]. However, it has been suggested that the high amount of bradykinin generated in the coagulation cascade leads to release of proteinase enzymes that destroy C5a, thus causing false negative results and that the formation of vasoactive mediators while the serum is being prepared leads to false positive results [26]. Because of these false negative and false positive results in the ASST, Asero et al. described the APST. They suggested that this *in vivo* test was more sensitive than ASST in patients with CAU [16], which, however, has not yet been approved by other authors [27, 28].

The APST was regarded to be positive according to different criteria in different studies. In some studies, the test result was taken as positive if an autologous plasma-induced wheal and flare had a diameter 3 mm greater than the control [16, 27]. In a study by Godse, only one unequivocal wheal-and-flare reaction with a wheal diameter of at least 1.5 mm greater than control was taken as a positive test result [29]. In previous studies, heparin, EDTA, and sodium citrate were used as anticoagulants while preparing plasma for the APST. However, it has been reported that heparin inhibits mast cell and basophil degranulation, thus leading to false negative results. EDTA has also been demonstrated to cause false positive results by leading to localized erythema and swelling at injection site in the patient and control groups. All these findings have pointed to sodium citrate as the most effective anticoagulant in the APST used for the detection of autoantibodies in patients with CAU [16]. For this reason, we used sodium citrate as the anticoagulant in this study. Of the 50 patients with CSU enrolled in this study, 31 (62%) had positive ASST and 21 (42%) had positive APST. In some studies, the prevalence of ASST positivity ranged from 42% to 68% whereas the prevalence of APST positivity varied from 14% to 97% [25, 27, 29]. No statistically significant differences were noted in ASST and APST positivity between the patient and control groups (ASST *P* < 0.001; APST *P* < 0.001). The compatibility of these two *in vivo* tests, which are used in the diagnosis of chronic autoimmune urticaria, was evaluated with Kappa test. And the relation between the two was found significant. In our paper; the tests were found to be %30 percent compatible with each other. That is why these two tests can be used in the diagnosis of autoimmune urticaria.

Some authors have attributed the high rate of positivity in the APST to the differences between the individuals performing the test, the absence of a standardized method for the assessment of the results, differences in patient selection, and differences in the presentation of the results [28, 30]. On the other hand, some authors have suggested that the APST is more sensitive than the ASST in patients with CAU [16, 25]. All relevant studies so far have reported that both the APST and ASST can be used for diagnostic purposes in patients with CAU [16, 25, 27, 29].

Previous studies have emphasized that antithyroid antibodies (anti-TPO, in particular) and abnormal thyroid function tests are more common in patients with CAU [31, 32]. Even though the incidence of antithyroid antibodies has been reported to range from 15% to 24% in chronic urticaria in the literature, the frequency of antithyroid autoantibody positivity has recently been reported to be 27.7% [33]. In the present study, 7 (14%) patients with CSU had positive anti-TG antibodies and 6 (12%) had positive anti-TPO antibodies. Three (6%) patients in the control group had positive anti-TG antibodies and 2 (4%) patients had positive anti-TPO antibodies. No significant differences were found in thyroid autoimmunity between the patients with CSU and the control group in this study. Additionally, we investigated whether there was a relationship between antithyroid antibody positivity and the ASST and APST in this study. No statistically significant differences were noted in the relationship between thyroid autoimmunity and ASST and APST positivity and negativity in patients with CU.

## 5. Conclusion

We consider that the ASST and APST can both be used as *in vivo* tests for the assessment of autoimmunity in the etiology of chronic urticaria, and that thyroid autoantibodies should be investigated even though thyroid function tests reveal normal results. 

## Figures and Tables

**Table 1:** The distribution of the patients and controls according to anti-TG antibodies, anti-TPO antibodies, and TSH levels.

	Patient (*n* = 50)				Control (*n* = 50)				*P*
	Yes		No		Yes		No		
	*n*	%	*n*	%	*n*	%	*n*	%	
Anti-TG	7	14	43	86	3	6	47	94	0.317
Anti-TPO	6	12	44	88	2	4	48	96	0.269
TSH									
Hypothyroidism	3	6	47	94	2	4	48	96	1,000
Euthyroidism	47	94	3	6	48	96	2	4	

Anti-TG: antithyroglobulin antibodies; Anti-TPO: antithyroid peroxidase antibodies; TSH: thyroid stimulating hormone.

**Table 2:** The distribution of ASST and APST results of the patient group.

	APST		Total
	Positive	Negative	
ASST			
Positive	14	17	31
Negative	7	12	19

Total	21	29	50

ASST: autologous serum skin test; APST: autologous plasma skin test.

**Table 3:** The distribution of ASST and APST results of the control group.

	APST		Total
	Positive	Negative	
ASST			
Positive	1	4	5
Negative	2	43	45

Total	3	47	50

ASST: autologous serum skin test; APST: autologous plasma skin test.

**Table 4:** The clinical characteristics of the patients according to ASST and APST responses.

	ASST					APST				
	Positive		Negative		*P*	Positive		Negative		*P*
Duration of disease (year)	2.97 ± 5.60		3.02 ± 4.20		0.970	3.63 ± 6.64		2.52 ± 3.60		0.449
Duration of papules (hour)	5.12 ± 4.87		7.52 ± 4.41		0.087	5.59 ± 4.31		6.36 ± 5.18		0.583
Interval between attacks (day)	2.0 ± 0.89		1.5 ± 0.69		0.060	2.0 ± 0.79		2.0 ± 0.89		0.496

	*n*	%	*n*	%		*n*	%	*n*	%	

Angioedema										
(+)	16	52	10	53	1.000	14	67	12	41	0.139
(−)	15	48	9	47		7	33	17	59	

ASST: autologous serum skin test; APST: autologous plasma skin test.

**Table 5:** The relationship between thyroid autoantibodies and ASST and APST results of the patients.

	ASST					APST				
	Positive		Negative		*P*	Positive		Negative		*P*
	*n*	%	*n*	%		*n*	%	*n*	%	
Anti-TG										
(+)	5	16	2	10.5	0.695	4	19	3	10	0.434
(−)	26	84	17	89.5		17	81	26	90	
Anti-TPO										
(+)	4	13	2	10.5	1.000	3	14	3	10	0.686
(−)	27	87	17	89.5		18	86	26	90	
TSH										
Hipothyroidism	0	0	3	16	**0.049**	0	0	3	10	0.254
Euthyroidism	31	100	26	84		21	100	26	90	

Anti-TG: antithyroglobulin antibodies; Anti-TPO: antithyroid peroxidase antibodies; TSH: thyroid stimulating hormone; ASST: autologous serum skin test; APST: autologous plasma skin test.

**Table 6:** The relationship between thyroid autoantibodies and ASST and APST results of the control group.

	ASST					APST				
	Positive		Negative		*P*	Positive		Negative		*P*
	*n*	%	*n*	%		*n*	%	*n*	%	
Anti-TG										
(+)	1	20	2	4.5	0.276	0	0	3	6	1.000
(−)	4	80	43	95.5		3	100	44	94	
Anti-TPO										
(+)	0	0	2	4.5	1.000	1	33.3	1	2	0.118
(−)	5	100	43	95.5		2	66.7	46	98	
TSH										
Hypothyroidism	0	0	2	4.5	1.000	0	0	2	4	1.000
Euthyroidism	5	100	43	95.5		3	100	45	96	

Anti-TG: antithyroglobulin antibodies; Anti-TPO: antithyroid peroxidase antibodies; TSH: thyroid stimulating hormone; ASST: autologous serum skin test; APST: autologous plasma skin test.
